# The Surgical Removal of Impacted Third Molars

**Published:** 1901-01-15

**Authors:** J. W. Shaw


THE
### DENTAL REGISTER.

Vol. LV. January 15, 1901. No. 1.

### THE SURGICAL REMOVAL OF IMPACTED THIRD MOLARS.

BY DR. J. W. SHAW.

Reprinted from *Dental Cosmos*, October, 1900.

*Mr. President, Ladies and Gentlemen:*—My subject, as you may have noticed by our program, is on the surgical removal of impacted lower third molars.

By impacted lower third molars I mean those so-called wisdom teeth that can not be extracted by ordinary means, by the use of the Physick, the ordinary forceps, or an elevator, without first making way by the extraction of the tooth or teeth next anterior, these teeth being wedged between the ramus and the second molar tooth either by insufficient space or malposition. The cause of this trouble, no doubt, can be ascribed to civilization, to the crossing of the races and to inequalities of stature. I suppose our forefathers, when the chief occupation was the getting something to eat, paid little thought to their teeth, and had no especial need to worry about their so-called wisdom teeth, being blessed with jaws sufficiently large to accommodate the full number of teeth, with occasional supernumeraries. But there came a change. With the advance of civilization the struggle became more than for the mere maintenance of the body, going on to the storing up of knowledge and the acquirement and the holding of this world's goods.

So it became a question of "brain, not brawn." Their thought, then, not being directed solely to the procuring of food, but to the storing of knowledge and to competition and triumph over their neighbor, called for the exercise and development of their brain, with the consequent enlargement of the upper portion of the head and brain case.

With the development and education of the brain it became the easier to procure food, and the food being better prepared, or at least so prepared as to scarcely require the exercise of the jaws, inversely, and in harmony with the laws of nature—the development and growth of those portions of the body that have use, and the atrophy of those without use—with this lack of exercise of the function of mastication there came the consequent lessening and shortening of the jaw, resulting in the squaring up of the face and head, the head of civilized man.

Cases that might seem to belong to an advanced class of over-development may be occasionally noted where the cranium is out of all proportion to the lower part of the head, and with these narrow, undeveloped jaws the teeth of necessity must be cramped for space.

Then comes the question of intermarriage of the races, with their differences of facial and bony formation; and, lastly, this heterogeneous mixing in marriage without regard to nationality, kith or kin, a veritable jumble of inequalities. One a strapping six-footer, healthy and with noble face, with jaws mighty and teeth in place, the other a little rosebud, blessed with grace, with jaws and teeth to match her face.

And now the offspring. Well, that's different. Inheritance with them does not do so well. Perchance, inheriting the large jaws of the one parent and the small teeth of the other, or *vice versa*, would account for the wide spaces occasionally seen between the teeth, or the great crowding of the teeth in the jaws, which so frequently occasion the trouble which gives me an excuse for presenting this subject.

My attention was first called to this especial field in 1887, soon after graduation. A brother suffering with an impacted lower third molar, and having lost the first molar, the second molar seemed very valuable to him. Attempts had been made at extraction without success. He had been told that it would be absolutely necessary to sacrifice the molar next forward, from which he appealed.

I conceived the idea that it would be unnecessary to remove the second molar if the bone overlying and withholding the offending third molar could be cut away, so as to release it.

Begging an opportunity to put my theory into practice, anesthesia was induced with ether, and with engine and suitable burs the bone binding upon the tooth was cut away, and, with an elevator inserted under the forward lower edge, leverage upward and backward was applied and the tooth was out. From that time forward until the present I have never found it necessary to sacrifice a good tooth for the sake of removing an offending one, and I have had a goodly number of what might justly be termed bad cases.

Here and now I want to enter a protest against what seems to me to be a needless sacrifice of valuable teeth, for I frequently hear reports and see evidences of cases where such sacrifices have been made.

The operation is so simple, with such good results, that I see no reason why it should not be adopted.

The chief points to be considered are sepsis, the relation of tooth and parts to the inferior dental artery and nerve, with the branching artery, the mylohyoid, underlying the internal oblique ridge, care in not applying too great a force in dislodging the tooth, thus avoiding the possibility of a fracture at the angle of the jaw.

The instruments required consist of a dental or surgical engine, with suitable burs. I prefer the round and cone-shaped burs, in sizes Nos. 6 to 12, a gum lancet of the axe shape, an elevator, straight, with good-sized handle, mouth prop and siphon, and possibly suitable forceps for grasping and removing the tooth after it has been dislodged.

The operation consists, first, in locating the tooth with its relation to the ramus and mandible and its bearings on the second molar, with the arteries and nerve underlying, and soft tissue involved. Then, with anesthesia induced, preferaby with ether, the mouth prop in place, the gum or tissue overlying is divided and separated from the bone to allow free use of the bur. With suitable burs the alveolar process and bone impinging about the tooth are quickly burred away to a sufficient extend to allow the dislodgment of the tooth; the placing of the elevator at the proper point for leverage, upward and backward force applied, and success is yours.

The assistant so manipulating the siphon as to draw the blood and saliva directly from the seat of operation, allows the operator to proceed rapidly with the work.

The after-treatment consists in syringing away the cuttings of bone and the keeping of the wound clean until sufficiently healed to care for itself, of course making use of hydrogen dioxid, or any of the many antiseptics that may find favor with the operator.

DISCUSSION.

Dr. Faxon: I have just had a case of that kind. The position was not quite so horizontal as this one, but I

decided that the tooth, from the decay of it, was almost perpendicular. I burred away in the same manner, although I did not bur down on the front part of the tooth, in order to give my elevator a chance. I performed this under cocain. I should have refused to do it unless they allowed etherization. I have not done any extracting for six or seven years, and only undertook this case because the gentleman was an intimate friend of mine. I have found this the most difficult position from which to extract teeth.

Dr. D. H. Allis: I have had several cases in the last five years, one case especially which came to my office recently. The left lower third molar was imbedded deeper down in the jaw than is shown in these charts. The distal buccal cusp was just visible above the gum, presenting its articulating face exactly to the side of the second molar, leaving a space between the two teeth which formed a splendid breeding ground for bacteria. The second molar was perfectly sound, but on passing a broach down between the teeth I came to an exposed pulp. The tooth had been decaying in that position so long that the pulp was actually exposed, and gave the patient a severe toothache for a long time. This same operation was performed without taking out the second molar. The saving point I want to bring out is this: we know that the third molar roots turn backward, and therefore it must take a backward, upward motion to extract these teeth usually. I have done so by this method several times, and it has been so successful that I want to bring this process before you to-night. Suppose you do not want to put your patient under ether, or you do not want to take out the tooth. You place absorbent cotton rolls on either side of the tooth, keeping the space *perfectly dry* at the time of operating; then pack a red base plate gutta-percha filling or wedge firmly between the two teeth. This acts in two ways: It stops decay, and the nature of the substance, gutta-percha, is to bulge, and in so doing it turns these roots up, and they can be reached. This can be left packed in, and then replaced by another as often as once in four or six months, and you will find that the tooth will gradually right itself from this to an upright, serviceable position. I have seen cases where I had contemplated taking the third molar out, but, using this method, I have found that they could be left there, and they are in service to-day.

Dr. Shaw: Almost invariably the people who are suffering with such teeth put off the extraction of them until it is positively necessary. I have had some where I did not think it would be possible to straighten them up. These are ordinary cases which require this procedure. In reference to the gutta-percha, one would have to look sharply after it. I had a case recently of a woman whose face was badly swollen. The teeth seemed to be perfectly sound, but on close examination I found a piece of gutta-percha which had evidently slipped and got back down between the gum, the tooth and the bone. I removed it, syringed the place and it got along all right, but at some future time she expects to have the tooth removed. If you find the impacted teeth decaying in this manner, I think it is well to use cocain and take an exploring needle and find out the position of the tooth before you make any further advance. This can easily be done under cocain with suitable exploring points.

